# Supplementary material for: Density responses of lesser-studied carnivores to habitat and management strategies in southern Tanzania’s Ruaha-Rungwa landscape
Source: PLoS One. 2021 Mar 30;16(3):e0242293. doi: 10.1371/journal.pone.0242293 (PMC8009394; doi:10.1371/journal.pone.0242293)
Supplement: S1 Appendix — (PDF) [file pone.0242293.s001.pdf]

S1 Appendix: Individual identification and sexing

Individual identification was performed by visually inspecting coat markings. The lead author examined and named each record once, then carried out a second run to check each of them. A different observer subsequently verified identifications to minimise mismatches, and any photographs with uncertain identification were excluded from analysis. We started with the species presenting the most defined markings, i.e. serval, and compared the position of spots on individuals' flanks [56]. The method consisted of processing all the pictures coming from one camera station, in chronological order, and then going to the next camera station. Each identified individual was characterised by an ID number, its sex (if determinable), and a distinctive spot arrangement (indicated in red in the pictures). The comparison of the coat markings of individuals featuring in subsequent records with identified individuals extended over the whole flank, with the key marker acting as a starting point to ease the process. We repeated the operation with striped hyaena and aardwolf, but this time focusing on fore-quarters and hind-quarters and selecting several key markers.

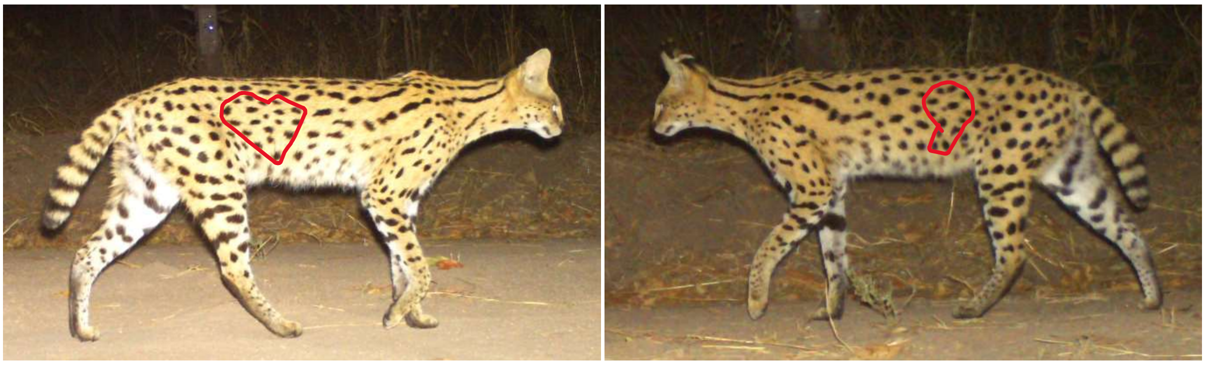

Core RNP *Acacia-Commiphora* grid – Serval Individual 01

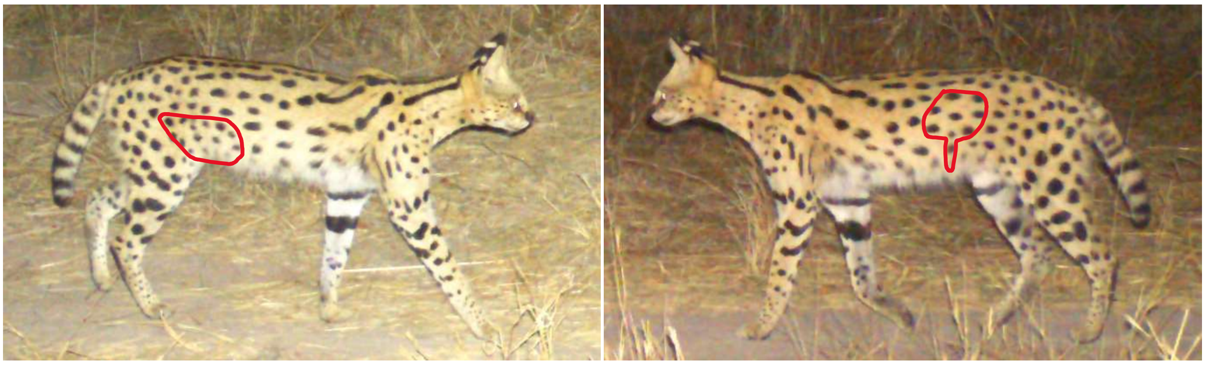

Core RNP *Acacia-Commiphora* grid – Serval Individual 04

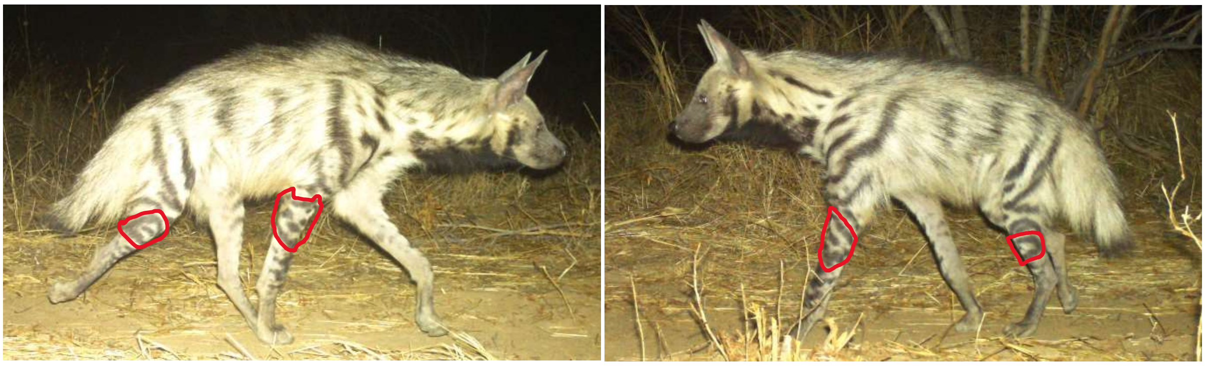

MBOMIPA WMA *Acacia-Commiphora* grid – Striped hyaena Individual 04

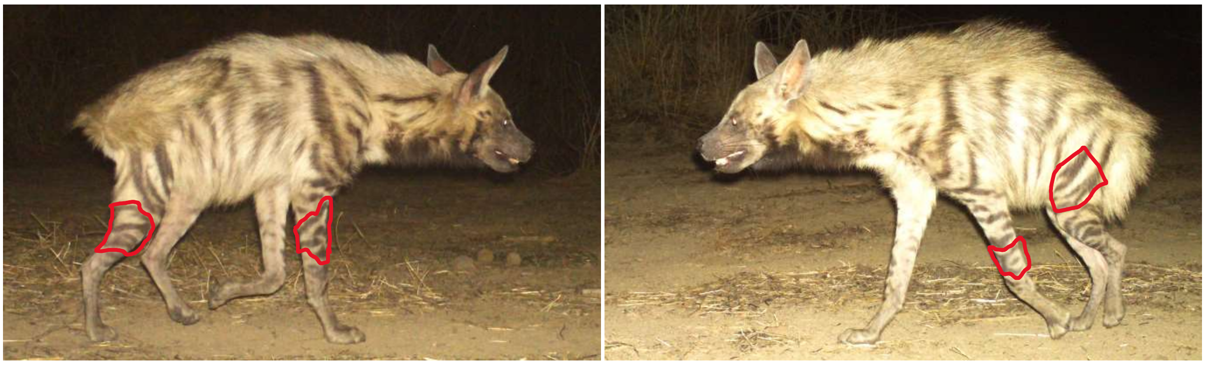

MBOMIPA WMA *Acacia-Commiphora* grid – Striped hyaena Individual 07

We sexed individuals based on the unobstructed view of external genitalia, late pregnancy signs such as weight gain and enlarged abdomen, or the presence of cubs.

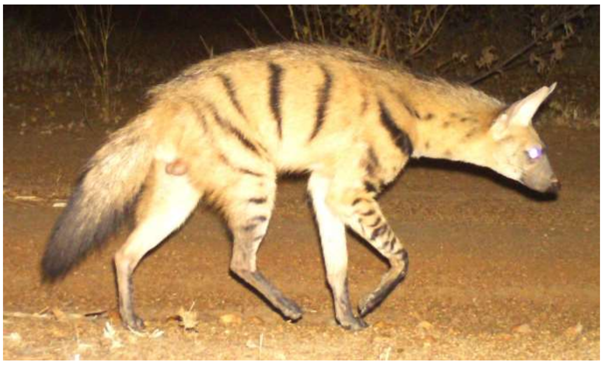

MBOMIPA WMA *Acacia-Commiphora* grid – Aardwolf Individual 22

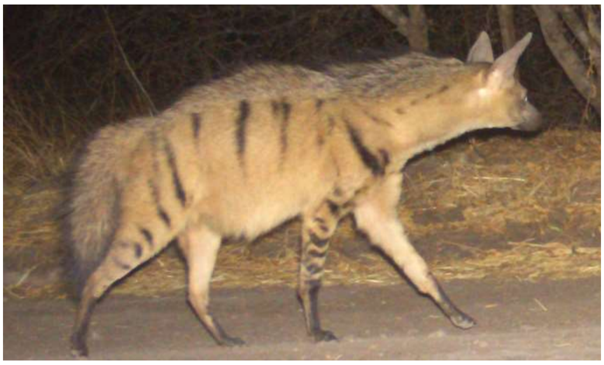

Core RNP *Acacia-Commiphora* grid – Aardwolf Individual 33
