# Supplementary material for: Density responses of lesser-studied carnivores to habitat and management strategies in southern Tanzania’s Ruaha-Rungwa landscape
Source: PLoS One. 2021 Mar 30;16(3):e0242293. doi: 10.1371/journal.pone.0242293 (PMC8009394; doi:10.1371/journal.pone.0242293)
Supplement: S2 Appendix — (PDF) [file pone.0242293.s002.pdf]

S2 Appendix: Capture histories

ID: individual identification SO: sampling occasion LOC_ID: Camera trap station ID

SERVAL									STRIPED HYAENA			AARDWOLF							
Core RNP *Acacia-Commiphora*			RNP miombo			MBOMIPA WMA *Acacia-Commiphora*			MBOMIPA WMA *Acacia-Commiphora*			Core RNP *Acacia-Commiphora*				MBOMIPA WMA *Acacia-Commiphora*			
ID	SO	LOC_ID	ID	SO	LOC_ID	ID	SO	LOC_ID	ID	SO	LOC_ID	ID	SO	LOC_ID	SEX	ID	SO	LOC_ID	SEX
1	15	4	1	4	4	1	5	4	1	46	1	1	4	37	M	1	36	7	M
1	19	4	1	7	4	1	16	36	1	53	28	1	11	37	M	1	45	7	M
1	20	4	1	9	4	1	21	2	2	53	25	1	15	2	M	1	60	7	M
1	21	4	1	13	4	2	56	3	2	53	30	1	15	37	M	3	14	25	NA
1	21	5	1	15	4	3	26	39	2	56	1	1	21	18	M	3	55	25	NA
1	23	4	1	17	4	3	34	40	2	59	2	1	21	19	M	4	15	19	NA
1	26	5	1	20	4	3	45	4	2	64	26	1	21	37	M	4	24	18	NA
1	28	4	1	23	4	3	57	4	2	68	23	1	25	37	M	4	35	15	NA
1	33	3	2	34	16	3	58	4	3	44	2	1	33	2	M	4	57	19	NA
1	69	4	2	57	4	4	44	4	4	11	25	1	51	18	M	6	4	8	M
1	71	4	2	70	12	5	9	8	4	12	26	1	51	19	M	6	18	8	M
4	14	32	3	26	4	5	26	8	4	38	11	1	55	19	M	6	22	9	M
4	14	40	5	53	4	5	51	9	5	14	12	1	64	37	M	6	27	8	M
4	24	13	6	34	5	6	36	11	5	33	12	1	65	3	M	6	28	9	M
4	29	32	6	47	5	6	38	11	6	11	19	1	66	26	M	6	30	8	M
4	31	9	8	85	5	7	50	15	6	27	13	1	81	3	M	6	38	8	M
4	35	9	9	2	7	7	53	22	6	42	13	2	30	17	NA	6	40	8	M
4	54	9	10	18	8	8	14	24	7	68	13	2	35	16	NA	6	41	8	M
4	64	40	11	5	9	8	20	30	8	42	14	3	24	9	NA	6	48	8	M
6	21	12	14	16	21	8	30	29	9	3	21	3	37	26	NA	6	49	8	M
7	10	14	16	50	12	8	58	24	9	4	17	3	42	3	NA	6	52	9	M
7	75	22	17	5	10	9	3	24	9	23	24	3	74	17	NA	6	58	8	M
8	17	16	17	72	24	9	15	24	9	27	21	4	25	6	M	7	26	8	NA
8	17	17				9	16	29	9	28	24	4	29	6	M	7	27	8	NA
10	21	19				9	22	23	9	30	15	4	30	4	M	8	47	14	NA
10	21	22				9	23	29	9	31	23	4	30	5	M	8	49	8	NA
11	8	26				9	23	31	9	33	24	4	55	6	M	8	49	9	NA
11	61	19				9	34	30	9	35	24	5	14	5	M	9	51	9	NA
12	30	22				9	45	23	9	37	21	5	26	5	M	9	52	8	NA
12	64	14				9	62	24	9	38	24	5	34	5	M	10	4	11	M
13	12	22				10	70	25	9	38	25	5	43	5	M	10	10	11	M
14	24	26							9	38	26	5	52	5	M	10	17	11	M
14	31	26							9	41	22	5	59	6	M	10	23	11	M
14	50	33							9	41	23	5	79	5	M	10	24	11	M
15	42	40							9	48	21	6	22	10	NA	10	26	11	M
15	43	32							9	51	25	6	29	40	NA	10	29	11	M
17	68	35							9	58	24	6	30	8	NA	10	31	11	M
18	13	28							11	66	17	6	56	40	NA	10	37	11	M
									12	25	26	6	66	40	NA	10	40	11	M

ID: individual identification SO: sampling occasion LOC_ID: Camera trap station ID

**SERVAL**									**STRIPED HYAENA**			**AARDWOLF**							
Core RNP *Acacia-Commiphora*			RNP miombo			MBOMIPA WMA *Acacia-Commiphora*			MBOMIPA WMA *Acacia-Commiphora*			Core RNP *Acacia-Commiphora*				MBOMIPA WMA *Acacia-Commiphora*			
ID	SO	LOC_ID	ID	SO	LOC_ID	ID	SO	LOC_ID	ID	SO	LOC_ID	ID	SO	LOC_ID	SEX	ID	SO	LOC_ID	SEX
									12	35	20	6	77	40	NA	10	50	11	M
									12	59	20	7	2	9	NA	10	52	11	M
									13	21	20	7	26	9	NA	10	53	11	M
												7	36	9	NA	10	56	11	M
												8	11	9	NA	10	58	11	M
												8	17	9	NA	10	59	11	M
												8	26	9	NA	10	63	11	M
												8	30	9	NA	11	21	11	NA
												8	48	9	NA	11	39	11	NA
												8	52	9	NA	11	41	11	NA
												8	55	9	NA	11	42	11	NA
												8	65	9	NA	11	45	11	NA
												9	13	10	M	11	47	11	NA
												9	17	10	M	12	23	11	M
												9	22	10	M	12	67	8	M
												9	25	10	M	13	12	13	M
												9	31	32	M	13	16	13	M
												9	39	10	M	13	34	13	M
												9	50	10	M	13	50	12	M
												9	51	32	M	13	53	13	M
												9	54	10	M	14	2	14	M
												9	58	10	M	14	11	14	M
												9	77	10	M	14	25	14	M
												10	13	11	NA	14	30	14	M
												10	18	11	NA	14	34	14	M
												10	20	10	NA	14	39	14	M
												10	26	10	NA	14	55	14	M
												10	40	10	NA	14	60	14	M
												10	45	11	NA	14	63	14	M
												10	48	10	NA	15	9	14	NA
												10	50	10	NA	15	37	14	NA
												10	52	10	NA	15	54	14	NA
												10	72	11	NA	15	64	14	NA
												10	73	10	NA	16	6	15	NA
												10	76	10	NA	16	9	27	NA
												10	82	10	NA	17	29	15	NA
												12	21	12	NA	17	46	16	NA
												12	25	45	NA	19	7	18	NA
												12	47	13	NA	19	9	19	NA

S2 Appendix: Capture histories

ID: individual identification SO: sampling occasion LOC_ID: Camera trap station ID

SERVAL									STRIPED HYAENA			AARDWOLF							
Core RNP *Acacia-Commiphora*			RNP miombo			MBOMIPA WMA *Acacia-Commiphora*			MBOMIPA WMA *Acacia-Commiphora*			Core RNP *Acacia-Commiphora*				MBOMIPA WMA *Acacia-Commiphora*			
ID	SO	LOC_ID	ID	SO	LOC_ID	ID	SO	LOC_ID	ID	SO	LOC_ID	ID	SO	LOC_ID	SEX	ID	SO	LOC_ID	SEX
												12	70	13	NA	19	29	19	NA
												13	24	19	NA	19	51	19	NA
												14	50	12	NA	19	69	19	NA
												14	74	22	NA	20	9	18	M
												14	75	22	NA	20	19	19	M
												14	77	22	NA	20	21	18	M
												14	82	22	NA	20	24	19	M
												15	10	13	NA	20	29	18	M
												15	59	13	NA	20	52	19	M
												16	18	13	NA	20	54	19	M
												17	14	22	M	20	63	19	M
												17	17	22	M	21	3	21	F
												17	20	22	M	21	13	21	F
												17	24	22	M	21	29	21	F
												17	33	22	M	21	56	21	F
												17	51	22	M	21	57	21	F
												17	56	25	M	22	13	24	M
												17	64	22	M	22	19	24	M
												17	77	22	M	22	23	24	M
												17	79	22	M	22	34	24	M
												18	5	17	NA	22	46	24	M
												18	16	17	NA	22	53	24	M
												18	19	17	NA	22	56	24	M
												18	20	17	NA	22	60	24	M
												18	34	17	NA	23	14	22	M
												18	64	17	NA	23	18	1	M
												18	81	17	NA	23	22	1	M
												19	7	17	M	23	27	22	M
												19	26	17	M	23	30	1	M
												19	38	17	M	23	38	23	M
												19	43	15	M	23	42	1	M
												19	67	17	M	23	45	22	M
												20	28	20	NA	23	47	1	M
												21	49	38	NA	23	51	1	M
												21	52	18	NA	23	56	23	M
												21	74	20	NA	23	64	1	M
												22	12	22	NA	24	16	22	NA
												22	29	22	NA	25	44	29	M
												22	34	22	NA	25	64	30	M

S2 Appendix: Capture histories

ID: individual identification SO: sampling occasion LOC_ID: Camera trap station ID

SERVAL									STRIPED HYAENA			AARDWOLF							
Core RNP *Acacia-Commiphora*			RNP miombo			MBOMIPA WMA *Acacia-Commiphora*			MBOMIPA WMA *Acacia-Commiphora*			Core RNP *Acacia-Commiphora*				MBOMIPA WMA *Acacia-Commiphora*			
ID	SO	LOC_ID	ID	SO	LOC_ID	ID	SO	LOC_ID	ID	SO	LOC_ID	ID	SO	LOC_ID	SEX	ID	SO	LOC_ID	SEX
												22	41	22	NA	26	5	26	M
												22	62	22	NA	26	7	27	M
												23	3	24	M	26	9	27	M
												23	14	23	M	26	10	26	M
												23	18	39	M	26	13	26	M
												23	30	39	M	26	16	14	M
												23	31	23	M	26	21	26	M
												23	33	24	M	26	23	26	M
												23	41	39	M	26	25	26	M
												23	45	39	M	26	45	26	M
												23	50	24	M	26	50	26	M
												23	51	39	M	26	67	5	M
												23	62	24	M	26	68	26	M
												23	70	23	M	27	13	26	M
												23	74	39	M	27	23	26	M
												23	76	24	M	27	27	26	M
												24	24	23	NA	27	37	26	M
												24	43	24	NA	27	42	26	M
												24	70	23	NA	27	60	26	M
												24	76	39	NA	27	62	26	M
												24	80	39	NA	28	7	18	M
												24	81	39	NA	28	7	27	M
												25	17	24	NA	28	10	27	M
												25	47	24	NA	28	24	27	M
												25	73	39	NA	28	38	27	M
												25	80	39	NA	28	44	27	M
												27	6	25	NA	28	52	27	M
												27	23	25	NA	29	9	27	NA
												28	48	23	NA	30	12	27	NA
												29	21	26	NA	30	18	27	NA
												30	11	27	M	30	21	27	NA
												30	18	27	M	30	46	27	NA
												30	19	27	M	30	52	27	NA
												30	20	28	M	30	55	27	NA
												30	27	27	M	33	5	30	NA
												30	27	28	M	33	20	29	NA
												30	29	28	M	33	22	29	NA
												30	33	27	M	33	24	30	NA
												30	39	28	M	33	28	30	NA

S2 Appendix: Capture histories

ID: individual identification SO: sampling occasion LOC_ID: Camera trap station ID

SERVAL									STRIPED HYAENA			AARDWOLF							
Core RNP *Acacia-Commiphora*			RNP miombo			MBOMIPA WMA *Acacia-Commiphora*			MBOMIPA WMA *Acacia-Commiphora*			Core RNP *Acacia-Commiphora*				MBOMIPA WMA *Acacia-Commiphora*			
ID	SO	LOC_ID	ID	SO	LOC_ID	ID	SO	LOC_ID	ID	SO	LOC_ID	ID	SO	LOC_ID	SEX	ID	SO	LOC_ID	SEX
												30	52	27	M	33	30	29	NA
												30	53	28	M	33	39	30	NA
												30	55	27	M	33	46	30	NA
												30	58	28	M	33	60	29	NA
												30	60	27	M	33	66	29	NA
												30	64	28	M	34	20	30	NA
												30	66	27	M	34	23	29	NA
												30	73	28	M	34	27	29	NA
												31	15	27	NA	35	30	30	NA
												31	18	27	NA	36	19	32	M
												31	28	27	NA	36	38	31	M
												31	42	27	NA	36	44	32	M
												31	53	27	NA	36	46	33	M
												31	57	27	NA	36	51	31	M
												31	60	27	NA	36	52	32	M
												31	67	27	NA	36	53	31	M
												31	72	27	NA	36	61	31	M
												32	55	43	NA	37	11	21	NA
												32	56	27	NA	37	28	21	NA
												33	3	28	F	37	51	21	NA
												33	5	28	F	38	68	8	NA
												33	13	28	F	39	39	18	NA
												33	25	28	F	39	47	17	NA
												33	33	28	F	39	53	18	NA
												33	67	28	F	41	50	5	NA
												33	82	28	F	41	52	14	NA
												34	21	28	NA	43	7	37	NA
												35	8	37	NA	44	10	24	F
												35	14	37	NA	44	13	24	F
												35	15	38	NA				
												35	18	37	NA				
												35	22	36	NA				
												35	27	37	NA				
												35	33	37	NA				
												35	35	37	NA				
												35	36	37	NA				
												35	43	37	NA				
												35	47	37	NA				
												35	48	37	NA				

ID: individual identification SO: sampling occasion LOC_ID: Camera trap station ID

SERVAL									STRIPED HYAENA			AARDWOLF							
Core RNP *Acacia-Commiphora*			RNP miombo			MBOMIPA WMA *Acacia-Commiphora*			MBOMIPA WMA *Acacia-Commiphora*			Core RNP *Acacia-Commiphora*				MBOMIPA WMA *Acacia-Commiphora*			
ID	SO	LOC_ID	ID	SO	LOC_ID	ID	SO	LOC_ID	ID	SO	LOC_ID	ID	SO	LOC_ID	SEX	ID	SO	LOC_ID	SEX
												35	50	37	NA				
												35	53	37	NA				
												35	55	37	NA				
												35	56	37	NA				
												35	60	37	NA				
												35	61	37	NA				
												35	63	37	NA				
												35	73	37	NA				
												35	74	37	NA				
												35	77	37	NA				
												35	82	37	NA				
												36	6	37	M				
												36	14	37	M				
												36	16	37	M				
												36	19	37	M				
												36	21	37	M				
												36	22	37	M				
												36	23	37	M				
												36	27	37	M				
												36	28	37	M				
												36	33	37	M				
												36	34	37	M				
												36	38	37	M				
												36	39	37	M				
												36	46	37	M				
												36	47	37	M				
												36	52	37	M				
												36	54	37	M				
												36	55	37	M				
												36	68	37	M				
												36	70	37	M				
												36	71	37	M				
												36	73	37	M				
												36	74	37	M				
												36	75	37	M				
												36	77	37	M				
												36	78	37	M				
												37	42	35	NA				
												37	49	40	NA				

S2 Appendix: Capture histories

ID: individual identification SO: sampling occasion LOC_ID: Camera trap station ID

**SERVAL**									**STRIPED HYAENA**			**AARDWOLF**							
Core RNP *Acacia-Commiphora*			RNP miombo			MBOMIPA WMA *Acacia-Commiphora*			MBOMIPA WMA *Acacia-Commiphora*			Core RNP *Acacia-Commiphora*				MBOMIPA WMA *Acacia-Commiphora*			
ID	SO	LOC_ID	ID	SO	LOC_ID	ID	SO	LOC_ID	ID	SO	LOC_ID	ID	SO	LOC_ID	SEX	ID	SO	LOC_ID	SEX
												37	51	35	NA				
												37	67	35	NA				
												37	83	10	NA				
												40	56	31	NA				
												40	66	31	NA				
												40	70	30	NA				
