# Supplementary material for: Density responses of lesser-studied carnivores to habitat and management strategies in southern Tanzania’s Ruaha-Rungwa landscape
Source: PLoS One. 2021 Mar 30;16(3):e0242293. doi: 10.1371/journal.pone.0242293 (PMC8009394; doi:10.1371/journal.pone.0242293)
Supplement: S3 Appendix — (PDF) [file pone.0242293.s003.pdf]

S3 Appendix: Trap layouts

Core RNP *Acacia-Commiphora*																					
**LOC_ID**	**X_Coord**	**Y_Coord**	**1**	**2**	**3**	**4**	**5**	**6**	**7**	**8**	**9**	**10**	**11**	**12**	**13**	**14**	**15**	**16**	**17**	**18**	**19**
**1**	**680198**	**9151369**	1	1	1	1	1	1	1	1	1	1	1	1	1	1	1	1	1	1	1
**2**	**682328**	**9152346**	1	1	1	1	1	1	1	1	1	1	1	1	1	1	1	1	1	1	1
**3**	**684718**	**9153207**	1	1	1	1	1	1	1	1	1	1	1	1	1	1	1	1	1	1	1
**4**	**686946**	**9153987**	1	1	1	1	1	1	1	1	1	1	1	1	1	1	1	1	1	1	1
**5**	**688751**	**9154848**	1	1	1	1	1	1	1	1	1	1	1	1	1	1	1	1	1	1	1
**6**	**690805**	**9156291**	1	1	1	1	1	1	1	1	1	1	1	1	1	1	1	1	1	1	1
**7**	**692587**	**9157188**	1	1	1	1	1	1	1	1	1	1	1	1	1	1	1	1	1	1	1
**8**	**695832**	**9157530**	1	1	1	1	1	1	1	1	1	1	1	1	1	1	1	1	1	1	1
**9**	**698050**	**9158612**	1	1	1	1	1	1	1	1	1	1	1	1	1	1	1	1	1	1	1
**10**	**697649**	**9155736**	1	1	1	1	1	1	1	1	1	1	1	1	1	1	1	1	1	1	1
**11**	**699747**	**9154421**	1	1	1	1	1	1	1	1	1	1	1	1	1	1	1	1	1	1	1
**12**	**698076**	**9151188**	1	1	1	1	1	1	1	1	1	1	1	1	1	1	1	1	1	1	1
**13**	**695173**	**9152197**	1	1	1	1	1	1	1	1	1	1	1	1	1	1	1	1	1	1	1
**14**	**693495**	**9150357**	1	1	1	1	1	1	1	1	1	1	1	1	1	1	1	1	1	1	1
**15**	**681462**	**9149527**	0	1	1	1	1	1	1	1	1	1	1	1	1	1	1	1	1	1	1
**16**	**684355**	**9148206**	0	1	1	1	1	1	1	1	1	1	1	1	1	1	1	1	1	1	1
**17**	**683417**	**9146385**	0	1	1	1	1	1	1	1	1	1	1	1	1	1	1	1	1	1	1
**18**	**686546**	**9148640**	0	1	1	1	1	1	1	1	1	1	1	1	1	1	1	1	1	1	1
**19**	**688860**	**9148003**	0	1	1	1	1	1	1	1	1	1	1	1	1	1	1	1	1	1	1
**20**	**690760**	**9146787**	0	1	1	1	1	1	1	1	1	1	1	1	1	1	1	1	1	1	1
**21**	**694333**	**9147041**	0	1	1	1	1	1	1	1	1	1	1	1	1	1	1	1	1	1	1
**22**	**695879**	**9148948**	0	1	1	1	1	1	1	1	1	1	1	1	1	1	1	1	1	1	1
**23**	**704702**	**9151645**	0	1	1	1	1	1	1	1	1	1	1	1	1	1	1	1	1	1	1
**24**	**701475**	**9151147**	0	1	1	1	1	1	1	1	1	1	1	1	1	1	1	1	1	1	1
**25**	**691443**	**9149809**	0	1	1	1	1	1	1	1	1	1	1	1	1	1	1	1	1	1	1
**26**	**688958**	**9149756**	0	1	1	1	1	1	1	1	1	1	1	1	1	1	1	1	1	1	1
**27**	**706425**	**9155293**	0	0	1	1	1	1	1	1	1	1	1	1	1	1	1	1	1	1	1
**28**	**706251**	**9158937**	0	0	1	1	1	1	1	1	1	1	1	1	1	1	1	1	1	1	1
**29**	**700748**	**9159243**	0	0	1	1	1	1	1	1	1	1	1	1	1	1	1	1	1	1	1
**30**	**703808**	**9157919**	0	0	1	1	1	1	1	1	1	1	1	1	1	1	1	1	1	1	1
**31**	**702712**	**9154855**	0	0	1	1	1	1	1	1	1	1	1	1	1	1	1	1	1	1	1
**32**	**694870**	**9156032**	0	0	1	1	1	1	1	1	1	1	1	1	1	1	1	1	1	1	1
**33**	**692076**	**9153044**	0	0	0	1	1	1	1	1	1	1	1	1	1	1	1	1	1	1	1
**34**	**689198**	**9152472**	0	0	0	1	1	1	1	1	1	1	1	1	1	1	1	1	1	1	1
**35**	**699493**	**9156857**	0	0	0	1	1	1	1	1	1	1	1	1	1	1	1	1	1	1	1
**36**	**685399**	**9151372**	0	0	0	1	1	1	1	1	1	1	1	1	1	1	1	1	1	1	1
**37**	**686084**	**9150161**	0	0	0	1	1	1	1	1	1	1	1	1	1	1	1	1	1	1	1
**38**	**683900**	**9150130**	0	0	0	1	1	1	1	1	1	1	1	1	1	1	1	1	1	1	1
**39**	**699987**	**9149051**	0	0	0	0	1	1	1	1	1	1	1	1	1	1	1	1	1	1	1
**40**	**694200**	**9154085**	0	0	0	0	0	0	0	0	0	0	0	0	1	1	1	1	1	1	1
**41**	**682130**	**9150150**	0	0	0	1	1	1	1	1	1	1	1	1	1	1	1	1	1	1	1
**42**	**698052**	**9150080**	0	0	0	0	0	1	1	1	1	1	1	1	1	1	1	1	1	1	1
**43**	**706553**	**9151716**	0	0	0	0	0	1	1	1	1	1	1	1	1	1	1	1	1	1	1
**44**	**698983**	**9156136**	0	0	0	0	0	0	0	0	0	0	0	0	0	0	0	0	0	0	0
**45**	**699145**	**9152745**	0	0	0	0	0	0	0	0	0	0	0	0	1	1	1	1	1	1	1

Core RNP *Acacia-Commiphora*																					
**LOC_ID**	**X_Coord**	**Y_Coord**	**20**	**21**	**22**	**23**	**24**	**25**	**26**	**27**	**28**	**29**	**30**	**31**	**32**	**33**	**34**	**35**	**36**	**37**	**38**
**1**	**680198**	**9151369**	1	1	1	1	1	1	1	1	1	1	1	1	1	1	1	1	1	1	1
**2**	**682328**	**9152346**	1	1	1	1	1	1	1	1	1	1	1	1	1	1	1	1	1	1	1
**3**	**684718**	**9153207**	1	1	1	1	1	1	1	1	1	1	1	1	1	1	1	1	1	1	1
**4**	**686946**	**9153987**	1	1	1	1	1	1	1	1	1	1	1	1	1	1	1	1	1	1	1
**5**	**688751**	**9154848**	1	1	1	1	1	1	1	1	1	1	1	1	1	1	1	1	1	1	1
**6**	**690805**	**9156291**	1	1	1	1	1	1	1	1	1	1	1	1	1	1	1	1	1	1	1
**7**	**692587**	**9157188**	1	1	1	1	1	1	1	1	1	1	1	1	1	1	1	1	1	1	1
**8**	**695832**	**9157530**	1	1	1	1	1	1	1	1	1	1	1	1	1	1	1	1	1	1	1
**9**	**698050**	**9158612**	1	1	1	1	1	1	1	1	1	1	1	1	1	1	1	1	1	1	1
**10**	**697649**	**9155736**	1	1	1	1	1	1	1	1	1	1	1	1	1	1	1	1	1	1	1
**11**	**699747**	**9154421**	1	1	1	1	1	1	1	1	1	1	1	1	1	1	1	1	1	1	1
**12**	**698076**	**9151188**	1	1	1	1	1	1	1	1	1	1	1	1	1	1	1	1	1	1	1
**13**	**695173**	**9152197**	1	1	1	1	1	1	1	1	1	1	1	1	1	1	1	1	1	1	1
**14**	**693495**	**9150357**	1	1	1	1	1	1	1	1	1	1	1	1	1	1	1	1	1	1	1
**15**	**681462**	**9149527**	1	1	1	1	1	1	1	1	1	1	1	1	1	1	1	1	1	1	1
**16**	**684355**	**9148206**	1	1	1	1	1	1	1	1	1	1	1	1	1	1	1	1	1	1	1
**17**	**683417**	**9146385**	1	1	1	1	1	1	1	1	1	1	1	1	1	1	1	1	1	1	1
**18**	**686546**	**9148640**	1	1	1	1	1	1	1	1	1	1	1	1	1	1	1	1	1	1	1
**19**	**688860**	**9148003**	1	1	1	1	1	1	1	1	1	1	1	1	1	1	1	1	1	1	1
**20**	**690760**	**9146787**	1	1	1	1	1	1	1	1	1	1	1	1	1	1	1	1	1	1	1
**21**	**694333**	**9147041**	1	1	1	1	1	1	1	1	1	1	1	1	1	1	1	1	1	1	1
**22**	**695879**	**9148948**	1	1	1	1	1	1	1	1	1	1	1	1	1	1	1	1	1	1	1
**23**	**704702**	**9151645**	1	1	1	1	1	1	1	1	1	1	1	1	1	1	1	1	1	1	1
**24**	**701475**	**9151147**	1	1	1	1	1	1	1	1	1	1	1	1	1	1	1	1	1	1	1
**25**	**691443**	**9149809**	1	1	1	1	1	1	1	1	1	1	1	1	1	1	1	1	1	1	1
**26**	**688958**	**9149756**	1	1	1	1	1	1	1	1	1	1	1	1	1	1	1	1	1	1	1
**27**	**706425**	**9155293**	1	1	1	1	1	1	1	1	1	1	1	1	1	1	1	1	1	1	1
**28**	**706251**	**9158937**	1	1	1	1	1	1	1	1	1	1	1	1	1	1	1	1	1	1	1
**29**	**700748**	**9159243**	1	1	1	1	1	1	1	1	1	1	1	1	1	1	1	1	1	1	1
**30**	**703808**	**9157919**	1	1	1	1	1	1	1	1	1	1	1	1	1	1	1	1	1	1	1
**31**	**702712**	**9154855**	1	1	1	1	1	1	1	1	1	1	1	1	1	1	1	1	1	1	1
**32**	**694870**	**9156032**	1	1	1	1	1	1	1	1	1	1	1	1	1	1	1	1	1	1	1
**33**	**692076**	**9153044**	1	1	1	1	1	1	1	1	1	1	1	1	1	1	1	1	1	1	1
**34**	**689198**	**9152472**	1	1	1	1	1	1	1	1	1	1	1	1	1	1	1	1	1	1	1
**35**	**699493**	**9156857**	1	1	1	1	1	1	1	1	1	1	1	1	1	1	1	1	1	1	1
**36**	**685399**	**9151372**	1	1	1	1	1	1	1	1	1	1	1	1	1	1	1	1	1	1	1
**37**	**686084**	**9150161**	1	1	1	1	1	1	1	1	1	1	1	1	1	1	1	1	1	1	1
**38**	**683900**	**9150130**	1	1	1	1	1	1	1	1	1	1	1	1	1	1	1	1	1	1	1
**39**	**699987**	**9149051**	1	1	1	1	1	1	1	1	1	1	1	1	1	1	1	1	1	1	1
**40**	**694200**	**9154085**	1	1	1	1	1	1	1	1	1	1	1	1	1	1	1	1	1	1	1
**41**	**682130**	**9150150**	1	1	1	1	1	1	1	1	1	1	1	1	1	1	1	1	1	1	1
**42**	**698052**	**9150080**	1	1	1	1	1	1	1	1	1	1	1	1	1	1	1	1	1	1	1
**43**	**706553**	**9151716**	1	1	1	1	1	1	1	1	1	1	1	1	1	1	1	1	1	1	1
**44**	**698983**	**9156136**	0	1	1	1	1	1	1	1	1	1	1	1	1	1	1	1	1	1	1
**45**	**699145**	**9152745**	1	1	1	1	1	1	1	1	1	1	1	1	1	1	1	1	1	1	1

Core RNP *Acacia-Commiphora*																					
LOC_ID	X_Coord	Y_Coord	39	40	41	42	43	44	45	46	47	48	49	50	51	52	53	54	55	56	57
1	680198	9151369	1	1	1	1	1	1	1	1	1	1	1	1	1	1	1	1	1	1	1
2	682328	9152346	1	1	1	1	1	1	1	1	1	1	1	1	1	1	1	1	1	1	1
3	684718	9153207	1	1	1	1	1	1	1	1	1	1	1	1	1	1	1	1	1	1	1
4	686946	9153987	1	1	1	1	1	1	1	1	1	1	1	1	1	1	1	1	1	1	1
5	688751	9154848	1	1	1	1	1	1	1	1	1	1	1	1	1	1	1	1	1	1	1
6	690805	9156291	1	1	1	1	1	1	1	1	1	1	1	1	1	1	1	1	1	1	1
7	692587	9157188	1	1	1	1	1	1	1	1	1	1	1	1	1	1	1	1	1	1	1
8	695832	9157530	1	1	1	1	1	1	1	1	1	1	1	1	1	1	1	1	1	1	1
9	698050	9158612	1	1	1	1	1	1	1	1	1	1	1	1	1	1	1	1	1	1	1
10	697649	9155736	1	1	1	1	1	1	1	1	1	1	1	1	1	1	1	1	1	1	1
11	699747	9154421	1	1	1	1	1	1	1	1	1	1	1	1	1	1	1	1	1	1	1
12	698076	9151188	1	1	1	1	1	1	1	1	1	1	1	1	1	1	1	1	1	1	1
13	695173	9152197	1	1	1	1	1	1	1	1	1	1	1	1	1	1	1	1	1	1	1
14	693495	9150357	1	1	1	1	1	1	1	1	1	1	1	1	1	1	1	1	1	1	1
15	681462	9149527	1	1	1	1	1	1	1	1	1	1	1	1	1	1	1	1	1	1	1
16	684355	9148206	1	1	1	1	1	1	1	1	1	1	1	1	1	1	1	1	1	1	1
17	683417	9146385	1	1	1	1	1	1	1	1	1	1	1	1	1	1	1	1	1	1	1
18	686546	9148640	1	1	1	1	1	1	1	1	1	1	1	1	1	1	1	1	1	1	1
19	688860	9148003	1	1	1	1	1	1	1	1	1	1	1	1	1	1	1	1	1	1	1
20	690760	9146787	1	1	1	1	1	1	1	1	1	1	1	1	1	1	1	1	1	1	1
21	694333	9147041	1	1	1	1	1	1	1	1	1	1	1	1	1	1	1	1	1	1	1
22	695879	9148948	1	1	1	1	1	1	1	1	1	1	1	1	1	1	1	1	1	1	1
23	704702	9151645	1	1	1	1	1	1	1	1	1	1	1	1	1	1	1	1	1	1	1
24	701475	9151147	1	1	1	1	1	1	1	1	1	1	1	1	1	1	1	1	1	1	1
25	691443	9149809	1	1	1	1	1	1	1	1	1	1	1	1	1	1	1	1	1	1	1
26	688958	9149756	1	1	1	1	1	1	1	1	1	1	1	1	1	1	1	1	1	1	1
27	706425	9155293	1	1	1	1	1	1	1	1	1	1	1	1	1	1	1	1	1	1	1
28	706251	9158937	1	1	1	1	1	1	1	1	1	1	1	1	1	1	1	1	1	1	1
29	700748	9159243	1	1	1	1	1	1	1	1	1	1	1	1	1	1	1	1	1	1	1
30	703808	9157919	1	1	1	1	1	1	1	1	1	1	1	1	1	1	1	1	1	1	1
31	702712	9154855	1	1	1	1	1	1	1	1	1	1	1	1	1	1	1	1	1	1	1
32	694870	9156032	1	1	1	1	1	1	1	1	1	1	1	1	1	1	1	1	1	1	1
33	692076	9153044	1	1	1	1	1	1	1	1	1	1	1	1	1	1	1	1	1	1	1
34	689198	9152472	1	1	1	1	1	1	1	1	1	1	1	1	1	1	1	1	1	1	1
35	699493	9156857	1	1	1	1	1	1	1	1	1	1	1	1	1	1	1	1	1	1	1
36	685399	9151372	1	1	1	1	1	1	1	1	1	1	1	1	1	1	1	1	1	1	1
37	686084	9150161	1	1	1	1	1	1	1	1	1	1	1	1	1	1	1	1	1	1	1
38	683900	9150130	1	1	1	1	1	1	1	1	1	1	1	1	1	1	1	1	1	1	1
39	699987	9149051	1	1	1	1	1	1	1	1	1	1	1	1	1	1	1	1	1	1	1
40	694200	9154085	1	1	1	1	1	1	1	1	1	1	1	1	1	1	1	1	1	1	1
41	682130	9150150	1	1	1	1	1	1	1	1	1	1	1	1	1	1	1	1	1	1	1
42	698052	9150080	1	1	1	1	1	1	1	1	1	1	1	1	1	1	1	1	1	1	1
43	706553	9151716	1	1	1	1	1	1	1	1	1	1	1	1	1	1	1	1	1	1	1
44	698983	9156136	1	1	1	1	1	1	1	1	1	1	1	1	1	1	1	1	1	1	1
45	699145	9152745	1	1	1	1	1	1	1	1	1	1	1	1	1	1	1	1	1	1	1

Core RNP *Acacia-Commiphora*																					
LOC_ID	X_Coord	Y_Coord	58	59	60	61	62	63	64	65	66	67	68	69	70	71	72	73	74	75	76
1	680198	9151369	1	1	1	1	1	1	1	1	1	1	1	1	1	1	1	1	1	1	1
2	682328	9152346	1	1	1	1	1	1	1	1	1	1	1	1	1	1	1	1	1	1	1
3	684718	9153207	1	1	1	1	1	1	1	1	1	1	1	1	1	1	1	1	1	1	1
4	686946	9153987	1	1	1	1	1	1	1	1	1	1	1	1	1	1	1	1	1	1	1
5	688751	9154848	1	1	1	1	1	1	1	1	1	1	1	1	1	1	1	1	1	1	1
6	690805	9156291	1	1	1	1	1	1	1	1	1	1	1	1	1	1	1	1	1	1	1
7	692587	9157188	1	1	1	1	1	1	1	1	1	1	1	1	1	1	1	1	1	1	1
8	695832	9157530	1	1	1	1	1	1	1	1	1	1	1	1	1	1	1	1	1	1	1
9	698050	9158612	1	1	1	1	1	1	1	1	1	1	1	1	1	1	1	1	1	1	1
10	697649	9155736	1	1	1	1	1	1	1	1	1	1	1	1	1	1	1	1	1	1	1
11	699747	9154421	1	1	1	1	1	1	1	1	1	1	1	1	1	1	1	1	1	1	1
12	698076	9151188	1	1	1	1	1	1	1	1	1	1	1	1	1	1	1	1	1	1	1
13	695173	9152197	1	1	1	1	1	1	1	1	1	1	1	1	1	1	1	1	1	1	1
14	693495	9150357	1	1	1	1	1	1	1	1	1	1	1	1	1	1	1	1	1	1	1
15	681462	9149527	1	1	1	1	1	1	1	1	1	1	1	1	1	1	1	1	1	1	1
16	684355	9148206	1	1	1	1	1	1	1	1	1	1	1	1	1	1	1	1	1	1	1
17	683417	9146385	1	1	1	1	1	1	1	1	1	1	1	1	1	1	1	1	1	1	1
18	686546	9148640	1	1	1	1	1	1	1	1	1	1	1	1	1	1	1	1	1	1	1
19	688860	9148003	1	1	1	1	1	1	1	1	1	1	1	1	1	1	1	1	1	1	1
20	690760	9146787	1	1	1	1	1	1	1	1	1	1	1	1	1	1	1	1	1	1	1
21	694333	9147041	1	1	1	1	1	1	1	1	1	1	1	1	1	1	1	1	1	1	1
22	695879	9148948	1	1	1	1	1	1	1	1	1	1	1	1	1	1	1	1	1	1	1
23	704702	9151645	1	1	1	1	1	1	1	1	1	1	1	1	1	1	1	1	1	1	1
24	701475	9151147	1	1	1	1	1	1	1	1	1	1	1	1	1	1	1	1	1	1	1
25	691443	9149809	1	1	1	1	1	1	1	1	1	1	1	1	1	1	1	1	1	1	1
26	688958	9149756	1	1	1	1	1	1	1	1	1	1	1	1	1	1	1	1	1	1	1
27	706425	9155293	1	1	1	1	1	1	1	1	1	1	1	1	1	1	1	1	1	1	1
28	706251	9158937	1	1	1	1	1	1	1	1	1	1	1	1	1	1	1	1	1	1	1
29	700748	9159243	1	1	1	1	1	1	1	1	1	1	1	1	1	1	1	1	1	1	1
30	703808	9157919	1	1	1	1	1	1	1	1	1	1	1	1	1	1	1	1	1	1	1
31	702712	9154855	1	1	1	1	1	1	1	1	1	1	1	1	1	1	1	1	1	1	1
32	694870	9156032	1	1	1	1	1	1	1	1	1	1	1	1	1	1	1	1	1	1	1
33	692076	9153044	1	1	1	1	1	1	1	1	1	1	1	1	1	1	1	1	1	1	1
34	689198	9152472	1	1	1	1	1	1	1	1	1	1	1	1	1	1	1	1	1	1	1
35	699493	9156857	1	1	1	1	1	1	1	1	1	1	1	1	1	1	1	1	1	1	1
36	685399	9151372	1	1	1	1	1	1	1	1	1	1	1	1	1	1	1	1	1	1	1
37	686084	9150161	1	1	1	1	1	1	1	1	1	1	1	1	1	1	1	1	1	1	1
38	683900	9150130	1	1	1	1	1	1	1	1	1	1	1	1	1	1	1	1	1	1	1
39	699987	9149051	1	1	1	1	1	1	1	1	1	1	1	1	1	1	1	1	1	1	1
40	694200	9154085	1	1	1	1	1	1	1	1	1	1	1	1	1	1	1	1	1	1	1
41	682130	9150150	1	1	1	1	1	1	1	1	1	1	1	1	1	1	1	1	1	1	1
42	698052	9150080	1	1	1	1	1	1	1	1	1	1	1	1	1	1	1	1	1	1	1
43	706553	9151716	1	1	1	1	1	1	1	1	1	1	1	1	1	1	1	1	1	1	1
44	698983	9156136	1	1	1	1	1	1	1	1	1	1	1	1	1	1	1	1	1	1	1
45	699145	9152745	1	1	1	1	1	1	1	1	1	1	1	1	1	1	1	1	1	1	1

Core RNP *Acacia-Commiphora*											
**LOC_ID**	**X_Coord**	**Y_Coord**	**77**	**78**	**79**	**80**	**81**	**82**	**83**	**Croad**	**Ctype**
**1**	**680198**	**9151369**	1	1	1	1	1	1	0	/1	1
**2**	**682328**	**9152346**	1	1	1	1	1	1	0	/1	1
**3**	**684718**	**9153207**	1	1	1	1	1	1	0	/1	1
**4**	**686946**	**9153987**	1	1	1	1	1	1	0	/1	1
**5**	**688751**	**9154848**	1	1	1	1	1	1	0	/1	1
**6**	**690805**	**9156291**	1	1	1	1	1	1	0	/1	1
**7**	**692587**	**9157188**	1	1	1	1	1	1	0	/1	1
**8**	**695832**	**9157530**	1	1	1	1	1	1	0	/1	1
**9**	**698050**	**9158612**	1	1	1	1	1	1	0	/1	1
**10**	**697649**	**9155736**	1	1	1	1	1	1	1	/1	1
**11**	**699747**	**9154421**	1	1	1	1	1	1	1	/1	1
**12**	**698076**	**9151188**	1	1	1	1	1	1	1	/1	1
**13**	**695173**	**9152197**	1	1	1	1	1	1	1	/1	1
**14**	**693495**	**9150357**	1	1	1	1	1	1	0	/1	1
**15**	**681462**	**9149527**	1	1	1	1	1	1	0	/1	1
**16**	**684355**	**9148206**	1	1	1	1	1	1	0	/1	1
**17**	**683417**	**9146385**	1	1	1	1	1	1	0	/1	1
**18**	**686546**	**9148640**	1	1	1	1	1	1	0	/1	1
**19**	**688860**	**9148003**	1	1	1	1	1	1	0	/1	1
**20**	**690760**	**9146787**	1	1	1	1	1	1	0	/1	1
**21**	**694333**	**9147041**	1	1	1	1	1	1	0	/1	1
**22**	**695879**	**9148948**	1	1	1	1	1	1	0	/1	1
**23**	**704702**	**9151645**	1	1	1	1	1	1	0	/1	1
**24**	**701475**	**9151147**	1	1	1	1	1	1	1	/1	1
**25**	**691443**	**9149809**	1	1	1	1	1	1	0	/1	1
**26**	**688958**	**9149756**	1	1	1	1	1	1	0	/1	1
**27**	**706425**	**9155293**	1	1	1	1	1	1	0	/1	1
**28**	**706251**	**9158937**	1	1	1	1	1	1	0	/1	1
**29**	**700748**	**9159243**	1	1	1	1	1	1	0	/1	1
**30**	**703808**	**9157919**	1	1	1	1	1	1	1	/1	1
**31**	**702712**	**9154855**	1	1	1	1	1	1	1	/1	1
**32**	**694870**	**9156032**	1	1	1	1	1	1	1	/0	1
**33**	**692076**	**9153044**	1	1	1	1	1	1	1	/0	1
**34**	**689198**	**9152472**	1	1	1	1	1	1	1	/0	1
**35**	**699493**	**9156857**	1	1	1	1	1	1	1	/0	1
**36**	**685399**	**9151372**	1	1	1	1	1	1	0	/0	1
**37**	**686084**	**9150161**	1	1	1	1	1	1	0	/1	1
**38**	**683900**	**9150130**	1	1	1	1	1	1	0	/1	1
**39**	**699987**	**9149051**	1	1	1	1	1	1	0	/1	1
**40**	**694200**	**9154085**	1	1	1	1	1	1	1	/1	1
**41**	**682130**	**9150150**	1	1	1	1	1	1	0	/1	0
**42**	**698052**	**9150080**	1	1	1	1	1	1	0	/1	1
**43**	**706553**	**9151716**	1	1	1	1	1	1	0	/1	1
**44**	**698983**	**9156136**	1	1	1	1	1	1	1	/0	1
**45**	**699145**	**9152745**	1	1	1	1	1	1	1	/1	1

RNP miombo													
LOC_ID	X_Coord	Y_Coord	1	2	3	4	5	6	7	8	9	10	11
1	630233	9149763	1	1	1	1	1	1	1	1	1	1	1
2	633143	9148824	1	1	1	1	1	1	1	1	1	1	1
3	634550	9146768	1	1	1	1	1	1	1	1	1	1	1
4	636260	9143910	1	1	1	1	1	1	1	1	1	1	1
5	636968	9141251	1	1	1	1	1	1	1	1	1	1	1
6	635512	9139559	0	1	1	1	1	1	1	1	1	1	1
7	633774	9137626	0	1	1	1	1	1	1	1	1	1	1
8	625064	9149907	0	1	1	1	1	1	1	1	1	1	1
9	628349	9150382	0	1	1	1	1	1	1	1	1	1	1
10	638014	9139276	0	1	1	1	1	1	1	1	1	1	1
11	631643	9140312	0	1	1	1	1	1	1	1	1	1	1
12	627892	9152115	0	0	1	1	1	1	1	1	1	1	1
13	628735	9149383	0	0	1	1	1	1	1	1	1	1	1
14	631598	9147532	0	0	1	1	1	1	1	1	1	1	1
15	633811	9150056	0	0	1	1	1	1	1	1	1	1	1
16	636818	9146580	0	0	1	1	1	1	1	1	1	1	1
17	631948	9151181	0	0	0	1	1	1	1	1	1	1	1
18	633471	9144857	0	0	0	1	1	1	1	1	1	1	1
19	632622	9136217	0	0	0	1	1	1	1	1	1	1	1
20	636205	9135252	0	0	0	1	1	1	1	1	1	1	1
21	638282	9133403	0	0	0	1	1	1	1	1	1	1	1
22	640046	9138755	0	0	0	1	1	1	1	1	1	1	1
23	635319	9142736	0	0	0	0	0	0	0	0	0	0	0
24	634379	9140519	0	0	0	0	0	0	0	0	0	0	0
25	640442	9140951	0	0	0	0	0	0	0	0	0	0	0
26	636667	9144502	0	0	0	0	0	0	0	0	0	0	0

RNP miombo													
LOC_ID	X_Coord	Y_Coord	12	13	14	15	16	17	18	19	20	21	22
1	630233	9149763	1	1	1	1	1	1	1	1	1	1	1
2	633143	9148824	1	1	1	1	1	1	1	1	1	1	1
3	634550	9146768	1	1	1	1	1	1	1	1	1	1	1
4	636260	9143910	1	1	1	1	1	1	1	1	1	1	1
5	636968	9141251	1	1	1	1	1	1	1	1	1	1	1
6	635512	9139559	1	1	1	1	1	1	1	1	1	1	1
7	633774	9137626	1	1	1	1	1	1	1	1	1	1	1
8	625064	9149907	1	1	1	1	1	1	1	1	1	1	1
9	628349	9150382	1	1	1	1	1	1	1	1	1	1	1
10	638014	9139276	1	1	1	1	1	1	1	1	1	1	1
11	631643	9140312	1	1	1	1	1	1	1	1	1	1	1
12	627892	9152115	1	1	1	1	1	1	1	1	1	1	1
13	628735	9149383	1	1	1	1	1	1	1	1	1	1	1
14	631598	9147532	1	1	1	1	1	1	1	1	1	1	1
15	633811	9150056	1	1	1	1	1	1	1	1	1	1	1
16	636818	9146580	1	1	1	1	1	1	1	1	1	1	1
17	631948	9151181	1	1	1	1	1	1	1	1	1	1	1
18	633471	9144857	1	1	1	1	1	1	1	1	1	1	1
19	632622	9136217	1	1	1	1	1	1	1	1	1	1	1
20	636205	9135252	1	1	1	1	1	1	1	1	1	1	1
21	638282	9133403	1	1	1	1	1	1	1	1	1	1	1
22	640046	9138755	1	1	1	1	1	1	1	1	1	1	1
23	635319	9142736	0	0	0	0	0	0	0	0	0	0	0
24	634379	9140519	0	0	0	0	0	0	0	0	0	0	0
25	640442	9140951	0	0	0	0	0	0	0	0	0	0	0
26	636667	9144502	0	0	0	0	0	0	0	0	0	0	0

RNP miombo													
**LOC_ID**	**X_Coord**	**Y_Coord**	**23**	**24**	**25**	**26**	**27**	**28**	**29**	**30**	**31**	**32**	**33**
**1**	**630233**	**9149763**	1	1	1	1	1	1	1	1	1	1	1
**2**	**633143**	**9148824**	1	1	1	1	1	1	1	1	1	1	1
**3**	**634550**	**9146768**	1	1	1	1	1	1	1	1	1	1	1
**4**	**636260**	**9143910**	1	1	1	1	1	1	1	1	1	1	1
**5**	**636968**	**9141251**	1	1	1	1	1	1	1	1	1	1	1
**6**	**635512**	**9139559**	1	1	1	1	1	1	1	1	1	1	1
**7**	**633774**	**9137626**	1	1	1	1	1	1	1	1	1	1	1
**8**	**625064**	**9149907**	1	1	1	1	1	1	1	1	1	1	1
**9**	**628349**	**9150382**	1	1	1	1	1	1	1	1	1	1	1
**10**	**638014**	**9139276**	1	1	1	1	1	1	1	1	1	1	1
**11**	**631643**	**9140312**	1	1	1	1	1	1	1	1	1	1	1
**12**	**627892**	**9152115**	1	1	1	1	1	1	1	1	1	1	1
**13**	**628735**	**9149383**	1	1	1	1	1	1	1	1	1	1	1
**14**	**631598**	**9147532**	1	1	1	1	1	1	1	1	1	1	1
**15**	**633811**	**9150056**	1	1	1	1	1	1	1	1	1	1	1
**16**	**636818**	**9146580**	1	1	1	1	1	1	1	1	1	1	1
**17**	**631948**	**9151181**	1	1	1	1	1	1	1	1	1	1	1
**18**	**633471**	**9144857**	1	1	1	1	1	1	1	1	1	1	1
**19**	**632622**	**9136217**	1	1	1	1	1	1	1	1	1	1	1
**20**	**636205**	**9135252**	1	1	1	1	1	1	1	1	1	1	1
**21**	**638282**	**9133403**	1	1	1	1	1	1	1	1	1	1	1
**22**	**640046**	**9138755**	1	1	1	1	1	1	1	1	1	1	1
**23**	**635319**	**9142736**	0	0	0	0	0	1	1	1	1	1	1
**24**	**634379**	**9140519**	0	0	0	0	0	1	1	1	1	1	1
**25**	**640442**	**9140951**	0	0	0	0	0	1	1	1	1	1	1
**26**	**636667**	**9144502**	0	0	0	0	0	1	1	1	1	1	1

RNP miombo													
LOC_ID	X_Coord	Y_Coord	34	35	36	37	38	39	40	41	42	43	44
1	630233	9149763	1	1	1	1	1	1	1	1	1	1	1
2	633143	9148824	1	1	1	1	1	1	1	1	1	1	1
3	634550	9146768	1	1	1	1	1	1	1	1	1	1	1
4	636260	9143910	1	1	1	1	1	1	1	1	1	1	1
5	636968	9141251	1	1	1	1	1	1	1	1	1	1	1
6	635512	9139559	1	1	1	1	1	1	1	1	1	1	1
7	633774	9137626	1	1	1	1	1	1	1	1	1	1	1
8	625064	9149907	1	1	1	1	1	1	1	1	1	1	1
9	628349	9150382	1	1	1	1	1	1	1	1	1	1	1
10	638014	9139276	1	1	1	1	1	1	1	1	1	1	1
11	631643	9140312	1	1	1	1	1	1	1	1	1	1	1
12	627892	9152115	1	1	1	1	1	1	1	1	1	1	1
13	628735	9149383	1	1	1	1	1	1	1	1	1	1	1
14	631598	9147532	1	1	1	1	1	1	1	1	1	1	1
15	633811	9150056	1	1	1	1	1	1	1	1	1	1	1
16	636818	9146580	1	1	1	1	1	1	1	1	1	1	1
17	631948	9151181	1	1	1	1	1	1	1	1	1	1	1
18	633471	9144857	1	1	1	1	1	1	1	1	1	1	1
19	632622	9136217	1	1	1	1	1	1	1	1	1	1	1
20	636205	9135252	1	1	1	1	1	1	1	1	1	1	1
21	638282	9133403	1	1	1	1	1	1	1	1	1	1	1
22	640046	9138755	1	1	1	1	1	1	1	1	1	1	1
23	635319	9142736	1	1	1	1	1	1	1	1	1	1	1
24	634379	9140519	1	1	1	1	1	1	1	1	1	1	1
25	640442	9140951	1	1	1	1	1	1	1	1	1	1	1
26	636667	9144502	1	1	1	1	1	1	1	1	1	1	1

RNP miombo													
LOC_ID	X_Coord	Y_Coord	45	46	47	48	49	50	51	52	53	54	55
1	630233	9149763	1	1	1	1	1	1	1	1	1	1	1
2	633143	9148824	1	1	1	1	1	1	1	1	1	1	1
3	634550	9146768	1	1	1	1	1	1	1	1	1	1	1
4	636260	9143910	1	1	1	1	1	1	1	1	1	1	1
5	636968	9141251	1	1	1	1	1	1	1	1	1	1	1
6	635512	9139559	1	1	1	1	1	1	1	1	1	1	1
7	633774	9137626	1	1	1	1	1	1	1	1	1	1	1
8	625064	9149907	1	1	1	1	1	1	1	1	1	1	1
9	628349	9150382	1	1	1	1	1	1	1	1	1	1	1
10	638014	9139276	1	1	1	1	1	1	1	1	1	1	1
11	631643	9140312	1	1	1	1	1	1	1	1	1	1	1
12	627892	9152115	1	1	1	1	1	1	1	1	1	1	1
13	628735	9149383	1	1	1	1	1	1	1	1	1	1	1
14	631598	9147532	1	1	1	1	1	1	1	1	1	1	1
15	633811	9150056	1	1	1	1	1	1	1	1	1	1	1
16	636818	9146580	1	1	1	1	1	1	1	1	1	1	1
17	631948	9151181	1	1	1	1	1	1	1	1	1	1	1
18	633471	9144857	1	1	1	1	1	1	1	1	1	1	1
19	632622	9136217	1	1	1	1	1	1	1	1	1	1	1
20	636205	9135252	1	1	1	1	1	1	1	1	1	1	1
21	638282	9133403	1	1	1	1	1	1	1	1	1	1	1
22	640046	9138755	1	1	1	1	1	1	1	1	1	1	1
23	635319	9142736	1	1	1	1	1	1	1	1	1	1	1
24	634379	9140519	1	1	1	1	1	1	1	1	1	1	1
25	640442	9140951	1	1	1	1	1	1	1	1	1	1	1
26	636667	9144502	1	1	1	1	1	1	1	1	1	1	1

RNP miombo													
LOC_ID	X_Coord	Y_Coord	56	57	58	59	60	61	62	63	64	65	66
1	630233	9149763	1	1	1	1	1	1	1	1	1	1	1
2	633143	9148824	1	1	1	1	1	1	1	1	1	1	1
3	634550	9146768	1	1	1	1	1	1	1	1	1	1	1
4	636260	9143910	1	1	1	1	1	1	1	1	1	1	1
5	636968	9141251	1	1	1	1	1	1	1	1	1	1	1
6	635512	9139559	1	1	1	1	1	1	1	1	1	1	1
7	633774	9137626	1	1	1	1	1	1	1	1	1	1	1
8	625064	9149907	1	1	1	1	1	1	1	1	1	1	1
9	628349	9150382	1	1	1	1	1	1	1	1	1	1	1
10	638014	9139276	1	1	1	1	1	1	1	1	1	1	1
11	631643	9140312	1	1	1	1	1	1	1	1	1	1	1
12	627892	9152115	1	1	1	1	1	1	1	1	1	1	1
13	628735	9149383	1	1	1	1	1	1	1	1	1	1	1
14	631598	9147532	1	1	1	1	1	1	1	1	1	1	1
15	633811	9150056	1	1	1	1	1	1	1	1	1	1	1
16	636818	9146580	1	1	1	1	1	1	1	1	1	1	1
17	631948	9151181	1	1	1	1	1	1	1	1	1	1	1
18	633471	9144857	1	1	1	1	1	1	1	1	1	1	1
19	632622	9136217	1	1	1	1	1	1	1	1	1	1	1
20	636205	9135252	1	1	1	1	1	1	1	1	1	1	1
21	638282	9133403	1	1	1	1	1	1	1	1	1	1	1
22	640046	9138755	1	1	1	1	1	1	1	1	1	1	1
23	635319	9142736	1	1	1	1	1	1	1	1	1	1	1
24	634379	9140519	1	1	1	1	1	1	1	1	1	1	1
25	640442	9140951	1	1	1	1	1	1	1	1	1	1	1
26	636667	9144502	1	1	1	1	1	1	1	1	1	1	1

RNP miombo													
LOC_ID	X_Coord	Y_Coord	67	68	69	70	71	72	73	74	75	76	77
1	630233	9149763	1	1	1	1	1	1	1	1	1	1	1
2	633143	9148824	1	1	1	1	1	1	1	1	1	1	1
3	634550	9146768	1	1	1	1	1	1	1	1	1	1	1
4	636260	9143910	1	1	1	1	1	1	1	1	1	1	1
5	636968	9141251	1	1	1	1	1	1	1	1	1	1	1
6	635512	9139559	1	1	1	1	1	1	1	1	1	1	1
7	633774	9137626	1	1	1	1	1	1	1	1	1	1	1
8	625064	9149907	1	1	1	1	1	1	1	1	1	1	1
9	628349	9150382	1	1	1	1	1	1	1	1	1	1	1
10	638014	9139276	1	1	1	1	1	1	1	1	1	1	1
11	631643	9140312	1	1	1	1	1	1	1	1	1	1	1
12	627892	9152115	1	1	1	1	1	1	1	1	1	1	1
13	628735	9149383	1	1	1	1	1	1	1	1	1	1	1
14	631598	9147532	1	1	1	1	1	1	1	1	1	1	1
15	633811	9150056	1	1	1	1	1	1	1	1	1	1	1
16	636818	9146580	1	1	1	1	1	1	1	1	1	1	1
17	631948	9151181	1	1	1	1	1	1	1	1	1	1	1
18	633471	9144857	1	1	1	1	1	1	1	1	1	1	1
19	632622	9136217	1	1	1	1	1	1	1	1	1	1	1
20	636205	9135252	1	1	1	1	1	1	1	1	1	1	1
21	638282	9133403	1	1	1	1	1	1	1	1	1	1	1
22	640046	9138755	1	1	1	1	1	1	1	1	1	1	1
23	635319	9142736	1	1	1	1	1	1	1	1	1	1	1
24	634379	9140519	1	1	1	1	1	1	1	1	1	1	1
25	640442	9140951	1	1	1	1	1	1	1	1	1	1	1
26	636667	9144502	1	1	1	1	1	1	1	1	1	1	1

RNP miombo													
LOC_ID	X_Coord	Y_Coord	78	79	80	81	82	83	84	85	86	87	88
1	630233	9149763	1	1	1	1	1	1	1	1	1	1	1
2	633143	9148824	1	1	1	1	1	1	1	1	1	1	1
3	634550	9146768	1	1	1	1	1	1	1	1	1	1	1
4	636260	9143910	1	1	1	1	1	1	1	1	1	1	1
5	636968	9141251	1	1	1	1	1	1	1	1	1	1	1
6	635512	9139559	1	1	1	1	1	1	1	1	1	1	1
7	633774	9137626	1	1	1	1	1	1	1	1	1	1	1
8	625064	9149907	1	1	1	1	1	1	1	1	1	1	1
9	628349	9150382	1	1	1	1	1	1	1	1	1	1	1
10	638014	9139276	1	1	1	1	1	1	1	1	1	1	1
11	631643	9140312	1	1	1	1	1	1	1	1	1	1	1
12	627892	9152115	1	1	1	1	1	1	1	1	1	1	1
13	628735	9149383	1	1	1	1	1	1	1	1	1	1	1
14	631598	9147532	1	1	1	1	1	1	1	1	1	1	1
15	633811	9150056	1	1	1	1	1	1	1	1	1	1	1
16	636818	9146580	1	1	1	1	1	1	1	1	1	1	1
17	631948	9151181	1	1	1	1	1	1	1	1	1	1	1
18	633471	9144857	1	1	1	1	1	1	1	1	1	1	1
19	632622	9136217	1	1	1	1	1	1	1	1	1	1	1
20	636205	9135252	1	1	1	1	1	1	1	1	1	1	1
21	638282	9133403	1	1	1	1	1	1	1	1	1	1	1
22	640046	9138755	1	1	1	1	1	1	1	1	1	1	1
23	635319	9142736	1	1	1	1	1	1	1	1	1	1	1
24	634379	9140519	1	1	1	1	1	1	1	1	1	1	1
25	640442	9140951	1	1	1	1	1	1	1	1	1	1	1
26	636667	9144502	1	1	1	1	1	1	1	1	1	1	1

RNP miombo						
LOC_ID	X_Coord	Y_Coord	89	90	Croad	Ctype
1	630233	9149763	1	0	/1	1
2	633143	9148824	1	0	/0	1
3	634550	9146768	1	0	/1	1
4	636260	9143910	1	1	/1	1
5	636968	9141251	1	1	/1	1
6	635512	9139559	1	1	/0	1
7	633774	9137626	1	1	/1	1
8	625064	9149907	1	0	/1	1
9	628349	9150382	1	0	/1	1
10	638014	9139276	1	1	/1	1
11	631643	9140312	1	1	/1	1
12	627892	9152115	1	0	/0	1
13	628735	9149383	1	0	/0	1
14	631598	9147532	1	0	/0	1
15	633811	9150056	1	0	/0	1
16	636818	9146580	1	0	/0	1
17	631948	9151181	1	0	/0	1
18	633471	9144857	1	1	/0	1
19	632622	9136217	1	1	/1	1
20	636205	9135252	1	1	/1	1
21	638282	9133403	1	1	/1	1
22	640046	9138755	1	1	/0	1
23	635319	9142736	1	1	/0	1
24	634379	9140519	1	1	/0	1
25	640442	9140951	1	1	/0	1
26	636667	9144502	1	1	/0	0

MBOMIPA WMA
Acacia-Commiphora

LOC_ID	X_Coord	Y_Coord	1	2	3	4	5	6	7	8	9	10	11	12	13	14	15	16	17
1	752397	9161717	1	1	1	1	1	1	1	1	1	1	1	1	1	1	1	1	1
2	749627	9160676	1	1	1	1	1	1	1	1	1	1	1	1	1	1	1	1	1
3	746434	9159475	1	1	1	1	1	1	1	1	1	1	1	1	1	1	1	1	1
4	743487	9158265	1	1	1	1	1	1	1	1	1	1	1	1	1	1	1	1	1
5	746448	9170689	0	1	1	1	1	1	1	1	1	1	1	1	1	1	1	1	1
6	744704	9170147	0	1	1	1	1	1	1	1	1	1	1	1	1	1	1	1	1
7	742138	9170450	0	1	1	1	1	1	1	1	1	1	1	1	1	1	1	1	1
8	739030	9168086	0	1	1	1	1	1	1	1	1	1	1	1	1	1	1	1	1
9	736437	9166846	0	1	1	1	1	1	1	1	1	1	1	1	1	1	1	1	1
10	736990	9165312	0	1	1	1	1	1	1	1	1	1	1	1	1	1	1	1	1
11	741425	9168092	0	1	1	1	1	1	1	1	1	1	1	1	1	1	1	1	1
12	739691	9171039	0	1	1	1	1	1	1	1	1	1	1	1	1	1	1	1	1
13	736249	9170789	0	1	1	1	1	1	1	1	1	1	1	1	1	1	1	1	1
14	745716	9172878	0	1	1	1	1	1	1	1	1	1	1	1	1	1	1	1	1
15	750829	9171399	0	1	1	1	1	1	1	1	1	1	1	1	1	1	1	1	1
16	752133	9171261	0	1	1	1	1	1	1	1	1	1	1	1	1	1	1	1	1
17	751187	9175317	0	0	1	1	1	1	1	1	1	1	1	1	1	1	1	1	1
18	749703	9172972	0	0	1	1	1	1	1	1	1	1	1	1	1	1	1	1	1
19	751830	9173554	0	0	1	1	1	1	1	1	1	1	1	1	1	1	1	1	1
20	753182	9175415	0	0	1	1	1	1	1	1	1	1	1	1	1	1	1	1	1
21	750269	9168284	0	0	1	1	1	1	1	1	1	1	1	1	1	1	1	1	1
22	754843	9163262	0	0	1	1	1	1	1	1	1	1	1	1	1	1	1	1	1
23	752937	9164879	0	0	1	1	1	1	1	1	1	1	1	1	1	1	1	1	1
24	750807	9166346	0	0	1	1	1	1	1	1	1	1	1	1	1	1	1	1	1
25	748838	9168889	0	0	1	1	1	1	1	1	1	1	1	1	1	1	1	1	1
26	748044	9171392	0	0	1	1	1	1	1	1	1	1	1	1	1	1	1	1	1
27	748179	9173864	0	0	1	1	1	1	1	1	1	1	1	1	1	1	1	1	1
28	749801	9164168	0	0	0	1	1	1	1	1	1	1	1	1	1	1	1	1	1
29	747717	9165630	0	0	0	1	1	1	1	1	1	1	1	1	1	1	1	1	1
30	747613	9167327	0	0	0	1	1	1	1	1	1	1	1	1	1	1	1	1	1
31	744396	9166803	0	0	0	1	1	1	1	1	1	1	1	1	1	1	1	1	1
32	743917	9164199	0	0	0	1	1	1	1	1	1	1	1	1	1	1	1	1	1
33	742305	9164982	0	0	0	1	1	1	1	1	1	1	1	1	1	1	1	1	1
34	739965	9163818	0	0	0	1	1	1	1	1	1	1	1	1	1	1	1	1	1
35	740089	9161844	0	0	0	1	1	1	1	1	1	1	1	1	1	1	1	1	1
36	740299	9159339	0	0	0	1	1	1	1	1	1	1	1	1	1	1	1	1	1
37	746308	9161677	0	0	0	0	1	1	1	1	1	1	1	1	1	1	1	1	1
38	743447	9160490	0	0	0	0	1	1	1	1	1	1	1	1	1	1	1	1	1
39	740665	9157051	0	0	0	0	1	1	1	1	1	1	1	1	1	1	1	1	1
40	739600	9155332	0	0	0	0	1	1	1	1	1	1	1	1	1	1	1	1	1

MBOMIPA WMA *Acacia-Commiphora*																			
**LOC_ID**	**X_Coord**	**Y_Coord**	**18**	**19**	**20**	**21**	**22**	**23**	**24**	**25**	**26**	**27**	**28**	**29**	**30**	**31**	**32**	**33**	**34**
**1**	**752397**	**9161717**	1	1	1	1	1	1	1	1	1	1	1	1	1	1	1	1	1
**2**	**749627**	**9160676**	1	1	1	1	1	1	1	1	1	1	1	1	1	1	1	1	1
**3**	**746434**	**9159475**	1	1	1	1	1	1	1	1	1	1	1	1	1	1	1	1	1
**4**	**743487**	**9158265**	1	1	1	1	1	1	1	1	1	1	1	1	1	1	1	1	1
**5**	**746448**	**9170689**	1	1	1	1	1	1	1	1	1	1	1	1	1	1	1	1	1
**6**	**744704**	**9170147**	1	1	1	1	1	1	1	1	1	1	1	1	1	1	1	1	1
**7**	**742138**	**9170450**	1	1	1	1	1	1	1	1	1	1	1	1	1	1	1	1	1
**8**	**739030**	**9168086**	1	1	1	1	1	1	1	1	1	1	1	1	1	1	1	1	1
**9**	**736437**	**9166846**	1	1	1	1	1	1	1	1	1	1	1	1	1	1	1	1	1
**10**	**736990**	**9165312**	1	1	1	1	1	1	1	1	1	1	1	1	1	1	1	1	1
**11**	**741425**	**9168092**	1	1	1	1	1	1	1	1	1	1	1	1	1	1	1	1	1
**12**	**739691**	**9171039**	1	1	1	1	1	1	1	1	1	1	1	1	1	1	1	1	1
**13**	**736249**	**9170789**	1	1	1	1	1	1	1	1	1	1	1	1	1	1	1	1	1
**14**	**745716**	**9172878**	1	1	1	1	1	1	1	1	1	1	1	1	1	1	1	1	1
**15**	**750829**	**9171399**	1	1	1	1	1	1	1	1	1	1	1	1	1	1	1	1	1
**16**	**752133**	**9171261**	1	1	1	1	1	1	1	1	1	1	1	1	1	1	1	1	1
**17**	**751187**	**9175317**	1	1	1	1	1	1	1	1	1	1	1	1	1	1	1	1	1
**18**	**749703**	**9172972**	1	1	1	1	1	1	1	1	1	1	1	1	1	1	1	1	1
**19**	**751830**	**9173554**	1	1	1	1	1	1	1	1	1	1	1	1	1	1	1	1	1
**20**	**753182**	**9175415**	1	1	1	1	1	1	1	1	1	1	1	1	1	1	1	1	1
**21**	**750269**	**9168284**	1	1	1	1	1	1	1	1	1	1	1	1	1	1	1	1	1
**22**	**754843**	**9163262**	1	1	1	1	1	1	1	1	1	1	1	1	1	1	1	1	1
**23**	**752937**	**9164879**	1	1	1	1	1	1	1	1	1	1	1	1	1	1	1	1	1
**24**	**750807**	**9166346**	1	1	1	1	1	1	1	1	1	1	1	1	1	1	1	1	1
**25**	**748838**	**9168889**	1	1	1	1	1	1	1	1	1	1	1	1	1	1	1	1	1
**26**	**748044**	**9171392**	1	1	1	1	1	1	1	1	1	1	1	1	1	1	1	1	1
**27**	**748179**	**9173864**	1	1	1	1	1	1	1	1	1	1	1	1	1	1	1	1	1
**28**	**749801**	**9164168**	1	1	1	1	1	1	1	1	1	1	1	1	1	1	1	1	1
**29**	**747717**	**9165630**	1	1	1	1	1	1	1	1	1	1	1	1	1	1	1	1	1
**30**	**747613**	**9167327**	1	1	1	1	1	1	1	1	1	1	1	1	1	1	1	1	1
**31**	**744396**	**9166803**	1	1	1	1	1	1	1	1	1	1	1	1	1	1	1	1	1
**32**	**743917**	**9164199**	1	1	1	1	1	1	1	1	1	1	1	1	1	1	1	1	1
**33**	**742305**	**9164982**	1	1	1	1	1	1	1	1	1	1	1	1	1	1	1	1	1
**34**	**739965**	**9163818**	1	1	1	1	1	1	1	1	1	1	1	1	1	1	1	1	1
**35**	**740089**	**9161844**	1	1	1	1	1	1	1	1	1	1	1	1	1	1	1	1	1
**36**	**740299**	**9159339**	1	1	1	1	1	1	1	1	1	1	1	1	1	1	1	1	1
**37**	**746308**	**9161677**	1	1	1	1	1	1	1	1	1	1	1	1	1	1	1	1	1
**38**	**743447**	**9160490**	1	1	1	1	1	1	1	1	1	1	1	1	1	1	1	1	1
**39**	**740665**	**9157051**	1	1	1	1	1	1	1	1	1	1	1	1	1	1	1	1	1
**40**	**739600**	**9155332**	1	1	1	1	1	1	1	1	1	1	1	1	1	1	1	1	1

MBOMIPA WMA *Acacia-Commiphora*																			
**LOC_ID**	**X_Coord**	**Y_Coord**	**35**	**36**	**37**	**38**	**39**	**40**	**41**	**42**	**43**	**44**	**45**	**46**	**47**	**48**	**49**	**50**	**51**
**1**	**752397**	**9161717**	1	1	1	1	1	1	1	1	1	1	1	1	1	1	1	1	1
**2**	**749627**	**9160676**	1	1	1	1	1	1	1	1	1	1	1	1	1	1	1	1	1
**3**	**746434**	**9159475**	1	1	1	1	1	1	1	1	1	1	1	1	1	1	1	1	1
**4**	**743487**	**9158265**	1	1	1	1	1	1	1	1	1	1	1	1	1	1	1	1	1
**5**	**746448**	**9170689**	1	1	1	1	1	1	1	1	1	1	1	1	1	1	1	1	1
**6**	**744704**	**9170147**	1	1	1	1	1	1	1	1	1	1	1	1	1	1	1	1	1
**7**	**742138**	**9170450**	1	1	1	1	1	1	1	1	1	1	1	1	1	1	1	1	1
**8**	**739030**	**9168086**	1	1	1	1	1	1	1	1	1	1	1	1	1	1	1	1	1
**9**	**736437**	**9166846**	1	1	1	1	1	1	1	1	1	1	1	1	1	1	1	1	1
**10**	**736990**	**9165312**	1	1	1	1	1	1	1	1	1	1	1	1	1	1	1	1	1
**11**	**741425**	**9168092**	1	1	1	1	1	1	1	1	1	1	1	1	1	1	1	1	1
**12**	**739691**	**9171039**	1	1	1	1	1	1	1	1	1	1	1	1	1	1	1	1	1
**13**	**736249**	**9170789**	1	1	1	1	1	1	1	1	1	1	1	1	1	1	1	1	1
**14**	**745716**	**9172878**	1	1	1	1	1	1	1	1	1	1	1	1	1	1	1	1	1
**15**	**750829**	**9171399**	1	1	1	1	1	1	1	1	1	1	1	1	1	1	1	1	1
**16**	**752133**	**9171261**	1	1	1	1	1	1	1	1	1	1	1	1	1	1	1	1	1
**17**	**751187**	**9175317**	1	1	1	1	1	1	1	1	1	1	1	1	1	1	1	1	1
**18**	**749703**	**9172972**	1	1	1	1	1	1	1	1	1	1	1	1	1	1	1	1	1
**19**	**751830**	**9173554**	1	1	1	1	1	1	1	1	1	1	1	1	1	1	1	1	1
**20**	**753182**	**9175415**	1	1	1	1	1	1	1	1	1	1	1	1	1	1	1	1	1
**21**	**750269**	**9168284**	1	1	1	1	1	1	1	1	1	1	1	1	1	1	1	1	1
**22**	**754843**	**9163262**	1	1	1	1	1	1	1	1	1	1	1	1	1	1	1	1	1
**23**	**752937**	**9164879**	1	1	1	1	1	1	1	1	1	1	1	1	1	1	1	1	1
**24**	**750807**	**9166346**	1	1	1	1	1	1	1	1	1	1	1	1	1	1	1	1	1
**25**	**748838**	**9168889**	1	1	1	1	1	1	1	1	1	1	1	1	1	1	1	1	1
**26**	**748044**	**9171392**	1	1	1	1	1	1	1	1	1	1	1	1	1	1	1	1	1
**27**	**748179**	**9173864**	1	1	1	1	1	1	1	1	1	1	1	1	1	1	1	1	1
**28**	**749801**	**9164168**	1	1	1	1	1	1	1	1	1	1	1	1	1	1	1	1	1
**29**	**747717**	**9165630**	1	1	1	1	1	1	1	1	1	1	1	1	1	1	1	1	1
**30**	**747613**	**9167327**	1	1	1	1	1	1	1	1	1	1	1	1	1	1	1	1	1
**31**	**744396**	**9166803**	1	1	1	1	1	1	1	1	1	1	1	1	1	1	1	1	1
**32**	**743917**	**9164199**	1	1	1	1	1	1	1	1	1	1	1	1	1	1	1	1	1
**33**	**742305**	**9164982**	1	1	1	1	1	1	1	1	1	1	1	1	1	1	1	1	1
**34**	**739965**	**9163818**	1	1	1	1	1	1	1	1	1	1	1	1	1	1	1	1	1
**35**	**740089**	**9161844**	1	1	1	1	1	1	1	1	1	1	1	1	1	1	1	1	1
**36**	**740299**	**9159339**	1	1	1	1	1	1	1	1	1	1	1	1	1	1	1	1	1
**37**	**746308**	**9161677**	1	1	1	1	1	1	1	1	1	1	1	1	1	1	1	1	1
**38**	**743447**	**9160490**	1	1	1	1	1	1	1	1	1	1	1	1	1	1	1	1	1
**39**	**740665**	**9157051**	1	1	1	1	1	1	1	1	1	1	1	1	1	1	1	1	1
**40**	**739600**	**9155332**	1	1	1	1	1	1	1	1	1	1	1	1	1	1	1	1	1

**MBOMIPA WMA** *Acacia-Commiphora*																			
**LOC_ID**	**X_Coord**	**Y_Coord**	**52**	**53**	**54**	**55**	**56**	**57**	**58**	**59**	**60**	**61**	**62**	**63**	**64**	**65**	**66**	**67**	**68**
**1**	**752397**	**9161717**	1	1	1	1	1	1	1	1	1	1	1	1	1	1	1	1	1
**2**	**749627**	**9160676**	1	1	1	1	1	1	1	1	1	1	1	1	1	1	1	1	1
**3**	**746434**	**9159475**	1	1	1	1	1	1	1	1	1	1	1	1	1	1	1	1	1
**4**	**743487**	**9158265**	1	1	1	1	1	1	1	1	1	1	1	1	1	1	1	1	1
**5**	**746448**	**9170689**	1	1	1	1	1	1	1	1	1	1	1	1	1	1	1	1	1
**6**	**744704**	**9170147**	1	1	1	1	1	1	1	1	1	1	1	1	1	1	1	1	1
**7**	**742138**	**9170450**	1	1	1	1	1	1	1	1	1	1	1	1	1	1	1	1	1
**8**	**739030**	**9168086**	1	1	1	1	1	1	1	1	1	1	1	1	1	1	1	1	1
**9**	**736437**	**9166846**	1	1	1	1	1	1	1	1	1	1	1	1	1	1	1	1	1
**10**	**736990**	**9165312**	1	1	1	1	1	1	1	1	1	1	1	1	1	1	1	1	1
**11**	**741425**	**9168092**	1	1	1	1	1	1	1	1	1	1	1	1	1	1	1	1	1
**12**	**739691**	**9171039**	1	1	1	1	1	1	1	1	1	1	1	1	1	1	1	1	1
**13**	**736249**	**9170789**	1	1	1	1	1	1	1	1	1	1	1	1	1	1	1	1	1
**14**	**745716**	**9172878**	1	1	1	1	1	1	1	1	1	1	1	1	1	1	1	1	1
**15**	**750829**	**9171399**	1	1	1	1	1	1	1	1	1	1	1	1	1	1	1	1	1
**16**	**752133**	**9171261**	1	1	1	1	1	1	1	1	1	1	1	1	1	1	1	1	1
**17**	**751187**	**9175317**	1	1	1	1	1	1	1	1	1	1	1	1	1	1	1	1	1
**18**	**749703**	**9172972**	1	1	1	1	1	1	1	1	1	1	1	1	1	1	1	1	1
**19**	**751830**	**9173554**	1	1	1	1	1	1	1	1	1	1	1	1	1	1	1	1	1
**20**	**753182**	**9175415**	1	1	1	1	1	1	1	1	1	1	1	1	1	1	1	1	1
**21**	**750269**	**9168284**	1	1	1	1	1	1	1	1	1	1	1	1	1	1	1	1	1
**22**	**754843**	**9163262**	1	1	1	1	1	1	1	1	1	1	1	1	1	1	1	1	1
**23**	**752937**	**9164879**	1	1	1	1	1	1	1	1	1	1	1	1	1	1	1	1	1
**24**	**750807**	**9166346**	1	1	1	1	1	1	1	1	1	1	1	1	1	1	1	1	1
**25**	**748838**	**9168889**	1	1	1	1	1	1	1	1	1	1	1	1	1	1	1	1	1
**26**	**748044**	**9171392**	1	1	1	1	1	1	1	1	1	1	1	1	1	1	1	1	1
**27**	**748179**	**9173864**	1	1	1	1	1	1	1	1	1	1	1	1	1	1	1	1	1
**28**	**749801**	**9164168**	1	1	1	1	1	1	1	1	1	1	1	1	1	1	1	1	1
**29**	**747717**	**9165630**	1	1	1	1	1	1	1	1	1	1	1	1	1	1	1	1	1
**30**	**747613**	**9167327**	1	1	1	1	1	1	1	1	1	1	1	1	1	1	1	1	1
**31**	**744396**	**9166803**	1	1	1	1	1	1	1	1	1	1	1	1	1	1	1	1	1
**32**	**743917**	**9164199**	1	1	1	1	1	1	1	1	1	1	1	1	1	1	1	1	1
**33**	**742305**	**9164982**	1	1	1	1	1	1	1	1	1	1	1	1	1	1	1	1	1
**34**	**739965**	**9163818**	1	1	1	1	1	1	1	1	1	1	1	1	1	1	1	1	1
**35**	**740089**	**9161844**	1	1	1	1	1	1	1	1	1	1	1	1	1	1	1	1	1
**36**	**740299**	**9159339**	1	1	1	1	1	1	1	1	1	1	1	1	1	1	1	1	1
**37**	**746308**	**9161677**	1	1	1	1	1	1	1	1	1	1	1	1	1	1	1	1	1
**38**	**743447**	**9160490**	1	1	1	1	1	1	1	1	1	1	1	1	1	1	1	1	1
**39**	**740665**	**9157051**	1	1	1	1	1	1	1	1	1	1	1	1	1	1	1	1	1
**40**	**739600**	**9155332**	1	1	1	1	1	1	1	1	1	1	1	1	1	1	1	1	1

MBOMIPA WMA *Acacia-Commiphora*						
**LOC_ID**	**X_Coord**	**Y_Coord**	**69**	**70**	**Croad**	**Ctype**
1	752397	9161717	1	0	/1	0
2	749627	9160676	1	0	/1	1
3	746434	9159475	1	0	/1	0
4	743487	9158265	1	0	/1	0
5	746448	9170689	1	0	/1	1
6	744704	9170147	1	0	/1	1
7	742138	9170450	1	0	/1	1
8	739030	9168086	1	0	/1	1
9	736437	9166846	1	0	/1	1
10	736990	9165312	1	0	/0	1
11	741425	9168092	1	0	/0	1
12	739691	9171039	1	0	/0	1
13	736249	9170789	1	0	/0	1
14	745716	9172878	1	0	/1	1
15	750829	9171399	1	0	/1	1
16	752133	9171261	1	0	/0	1
17	751187	9175317	1	0	/1	1
18	749703	9172972	1	0	/1	1
19	751830	9173554	1	0	/1	1
20	753182	9175415	1	0	/1	1
21	750269	9168284	1	0	/1	1
22	754843	9163262	1	0	/1	1
23	752937	9164879	1	0	/1	1
24	750807	9166346	1	0	/1	1
25	748838	9168889	1	1	/1	1
26	748044	9171392	1	1	/1	1
27	748179	9173864	1	1	/1	1
28	749801	9164168	1	1	/0	1
29	747717	9165630	1	1	/0	1
30	747613	9167327	1	0	/1	1
31	744396	9166803	1	0	/1	1
32	743917	9164199	1	1	/0	1
33	742305	9164982	1	0	/1	1
34	739965	9163818	1	0	/1	1
35	740089	9161844	1	0	/1	1
36	740299	9159339	1	0	/1	1
37	746308	9161677	1	0	/0	1
38	743447	9160490	1	0	/0	1
39	740665	9157051	1	0	/1	0
40	739600	9155332	1	0	/1	0
