# Supplementary material for: Density responses of lesser-studied carnivores to habitat and management strategies in southern Tanzania’s Ruaha-Rungwa landscape
Source: PLoS One. 2021 Mar 30;16(3):e0242293. doi: 10.1371/journal.pone.0242293 (PMC8009394; doi:10.1371/journal.pone.0242293)
Supplement: S4 Appendix — (PDF) [file pone.0242293.s004.pdf]

S4 Appendix: Survey grid summary information

	Core RNP *Acacia-Commiphora*		RNP miombo	MBOMIPA WMA *Acacia-Commiphora*		
Survey duration (nights)	83		90	70		
Stations	45		26	40		
Trap nights	3,601		2,187	2,689		
Average spacing (km)	1.96		1.88	2.08		
Survey area (km^2)[1]	223		152	270		
	**Serval**	**Aardwolf**	**Serval**	**Serval**	**Aardwolf**	**Striped hyaena**
Number of pictures	81	643	59	86	476	125
Nb of pictures identified	70	523	48	77	385	91
Captures between 7pm - 6am	86.5%	98.8%	88.2%	82.9%	100%	98%
Flank with most captures	Left	Left	Right	Right	Left	Left
Individuals identified[2]	13	36	12	10	37	12
Female	1	1	0	0	2	0
Male	0	9	0	0	14	2
Sex unknown	12	26	12	10	21	10
Capture events[2]	38	240	23	31	185	42
Recapture rate[2,3]	69.2%	83.3%	33.3%	70%	86.5%	58.3%
Maximum movement (km)	9.02	14.04	11.72	9.42	11.79	15.84
Buffer width (km)	9	11	15	26	10	6
ML integration area (km^2)[4]	1047	1074	1883	3991	1173	700

[1] Area of the minimum convex polygon around all stations (does not include buffer)

[2] Based on the flank with most captures for each grid

[3] Percentage of identified individuals recaptured during the survey period

[4] Area of the minimum convex polygon around all stations including buffer
