# Supplementary material for: Density responses of lesser-studied carnivores to habitat and management strategies in southern Tanzania’s Ruaha-Rungwa landscape
Source: PLoS One. 2021 Mar 30;16(3):e0242293. doi: 10.1371/journal.pone.0242293 (PMC8009394; doi:10.1371/journal.pone.0242293)
Supplement: S5 Appendix — (PDF) [file pone.0242293.s005.pdf]

S5 Appendix: SECR model ranking

Model	nPar	LogLik	AICc	ΔAICc	AICc Wt
**Serval**					
**Core RNP – *Acacia-Commiphora***					
g0[.] σ[.]	3	-243.2113	495.089	0.000	0.5935
g0[b] σ[.]	4	-242.1878	497.376	2.287	0.1892
g0[bk] σ[.]	4	-242.4282	497.856	2.767	0.1488
g0[road] σ[.]	4	-243.2040	499.408	4.319	0.0685
**RNP – miombo**					
g0[road] σ[.]	4	-143.8776	301.469	0.000	0.9607
g0[.] σ[.]	3	-149.6443	308.289	6.820	0.0317
g0[b] σ[.]	4	-148.7227	311.160	9.691	0.0076
**MBOMIPA WMA – *Acacia-Commiphora***					
g0[.] σ[.]	3	-202.2945	414.589	0.000	0.6134
g0[road] σ[.]	4	-200.7411	417.482	2.893	0.1444
g0[b] σ[.]	4	-200.9592	417.918	3.329	0.1161
g0[bk] σ[.]	4	-201.5376	419.075	4.486	0.0651
g0[flash] σ[.]	4	-201.6024	419.205	4.616	0.0610
**Striped hyaena**					
**MBOMIPA WMA – *Acacia-Commiphora***					
g0[bk] σ[.]	4	-260.2405	534.195	0.000	0.5093
g0[.] σ[.]	3	-263.1278	535.256	1.061	0.2996
g0[b] σ[.]	4	-262.0198	537.754	3.559	0.0859
g0[road] σ[.]	4	-262.4954	538.705	4.510	0.0534
g0[flash] σ[.]	4	-262.5276	538.769	4.574	0.0517
**Aardwolf**					
**Core RNP – *Acacia-Commiphora***					
g0[bk] σ[.]	5	-1078.5530	2169.106	0.000	1.000
g0[road] σ[.]	5	-1096.8730	2205.745	36.639	0.000
g0[sex] σ[sex]	6	-1104.1150	2223.127	54.021	0.000
g0[.] σ[sex]	5	-1106.5100	2225.019	55.913	0.000
g0[sex] σ[.]	5	-1108.8340	2229.669	60.563	0.000
g0[b] σ[.]	5	-1113.9000	2239.800	70.694	0.000
g0[.] σ[.]	4	-1115.7990	2240.889	71.783	0.000

**MBOMIPA WMA – *Acacia-Commiphora***					
g0[sex] σ[.]	5	-838.9920	1689.919	0.000	0.5320
g0[.] σ[sex]	5	-839.7593	1691.454	1.535	0.2469
g0[sex] σ[sex]	6	-838.8898	1692.580	2.661	0.1406
g0[bk] σ[.]	5	-841.0557	1694.047	4.128	0.0675
g0[.] σ[.]	4	-844.4128	1698.076	8.157	0.0090
g0[b] σ[.]	5	-843.8918	1699.719	9.800	0.0040
g0[road] σ[.]	5	-844.2994	1700.534	10.615	0.0000

Rows shaded in light grey indicate models with substantial empirical support
(ΔAICc < 2 from the top-ranked model)
g0: detection probability at home range centre
σ: distance parameter related to home range size
g0[.] σ[.]: g0 and sigma constant
g0[b] σ[.]: permanent & global step change after first detection for g0
g0[bk] σ[.]: permanent & site-specific step change after first detection for g0
g0[road] σ[.]: g0 varies with station location (on-road vs off-road)
g0[flash] σ[.]: g0 varies with station type of flash (xenon vs LED flash)
g0[sex] σ[.]: g0 varies with sex
g0[.] σ[sex]: σ varies with sex
g0[sex] σ[sex]: g0 and σ vary with sex
